# Association Between Physical Exercise Interventions Participation and Functional Capacity in Individuals with Type 2 Diabetes: A Systematic Review and Meta-Analysis of Controlled Trials

**DOI:** 10.1186/s40798-022-00422-1

**Published:** 2022-03-04

**Authors:** Lucinéia Orsolin Pfeifer, Angélica Trevisan De Nardi, Larissa Xavier Neves da Silva, Cíntia Ehlers Botton, Daniela Meirelles do Nascimento, Juliana Lopes Teodoro, Beatriz D. Schaan, Daniel Umpierre

**Affiliations:** 1 Exercise Pathophysiology Research Laboratory, Graduate Program in Cardiology and Cardiovascular Sciences, Universidade Federal do Rio Grande do Sul, Porto Alegre, RS Brazil; 2 Exercise Pathophysiology Research Laboratory, Clinical Research Center, Hospital de Clínicas de Porto Alegre, Rua Ramiro Barcelos, 2350, Porto Alegre, RS 21301 Brazil; 3 Exercise Research Laboratory, Graduate Program in Human Movement Science, Universidade Federal do Rio Grande do Sul, Porto Alegre, RS Brazil; 4 Universidade Federal do Rio Grande do Sul, Porto Alegre, RS Brazil

**Keywords:** Functional capacity, Structured exercise training, Type 2 diabetes, Systematic review, Meta-analysis

## Abstract

**Background:**

The prevalence of type 2 diabetes mellitus increases with age, and people with type 2 diabetes are more affected by reductions in functional performance. Although exercise interventions are recommended for people with diabetes, it is relevant to assess the effects of different training modes on the available functional outcomes. Therefore, our purpose was to systematically assess the effect of different physical exercise modalities in patients with type 2 diabetes with an average age of 45 years or older on outcomes used to measure functional capacity.

**Methods:**

A systematic review and meta-analysis of controlled trials was conducted. Seven databases were searched from January 1987 to December 2021 (PubMed, Physiotherapy Evidence Database, Cochrane Library, SPORTDiscus, and in grey literature: Open Grey and Google Scholar). Eligible studies should last 8 weeks or longer, comparing structured exercise training and non-exercise control for one out of six pre-specified functional capacity outcomes (Timed Up and Go test, chair stands, walking performance, upper-limb muscle strength, lower-limb muscle strength, physical fitness parameter), in patients with type 2 diabetes, aged ≥ 45 years. The risk of bias was assessed with the Downs & Black checklist. Pooled mean differences were calculated using a random-effects model, followed by sensitivity and meta-regression analyses.

**Results:**

Of 18,112 references retrieved, 29 trials (1557 patients) were included. Among these, 13 studies used aerobic training, 6 studies used combined training, 4 studies used resistance training, 3 studies had multiple intervention arms and 3 studies used other types of training. Exercise training was associated with an increase in functional capacity outcomes, as reflected by changes in 6-min walk test (*n* = 8) [51.6 m; 95% CI 7.6% to 95.6%; I^2^ 92%], one-repetition maximum leg-press (*n* = 3) [18.0 kg; 95% CI 4.0% to 31.9%; I^2^ 0%], and maximum oxygen consumption (VO_2max_) (*n* = 20) [2.41 mL/kg·min; 95% CI 1.89% to 2.92%; I^2^ 100%] compared with control groups. In sensitivity and subgroup analyses using VO_2max_ as outcome and stratified by type of study (randomized and non-randomized controlled clinical trials), duration of diabetes diagnosis, and sex, we observed overlapping confidence intervals. Meta-regression showed no association between glycated hemoglobin (HbA1C) levels and VO_2max_ [*p* = 0.34; I^2^ 99.6%; *R*^2^ = 2.6%]. In addition, the quality of the included studies was mostly low.

**Conclusion:**

The results indicate that structured physical exercise programs might improve functional capacity in patients with type 2 diabetes, except for the upper-limb muscle strength. However, we could not identify potential effect predictors associated with directional summary estimates.

*Trial registration* This systematic review was registered in the PROSPERO international prospective register of systematic reviews (CRD42020162467); date of registration: 12/15/2019. The review protocol is hosted at the Open Science Framework (OSF) (Preprint https://doi.org/10.31219/osf.io/kpg2m).

**Supplementary Information:**

The online version contains supplementary material available at 10.1186/s40798-022-00422-1.

## Key Points

- Structured physical exercise lasting 8 weeks or more is associated with increases in functional capacity in people at an average age of 45 years or older with type 2 diabetes.
- The additional analyses related to sex, duration of disease diagnosis, and type of study were inconclusive in this synthesis.
- Future research is warranted investigating the effect of structured exercise on younger populations as well and in people with diabetes who are often excluded from trials. Furthermore, studies with primary outcomes of functional capacity are needed.

## Background

Diabetes mellitus is an increasingly prevalent chronic-degenerative disease, generating a burden on public health. In 2019, the International Diabetes Federation estimated that 1 out of 11 adults in the world population aged 20 to 79 lived with diabetes, equivalent to 463 million people [1]. Notably, type 2 diabetes mellitus is a common disease in older adults [1], who also experience reductions in neuromuscular function, muscle mass, muscle strength, and motor performance [2]. Compared with non-diabetic individuals, older adults with diabetes have accelerated loss of muscle mass, muscle strength, muscle quality, and neural function [3–5], worsening the performance in functional tests [3, 6], contributing to a marked increase in physical disability and frailty risks in this population [7, 8]. The risk of physical disability for adult people with diabetes increases by about 50 to 80% compared with age-matched individuals without diabetes [8].

Functional capacity has multidimensional features and is considered the individual's ability to perform instrumental activities in their daily lives, sustaining their autonomy. Functional performance measures reflect a particular aspect of physical functioning by using mostly objective and predetermined criteria, that is, in which individuals are asked to actually perform specific tasks and are evaluated using standardized criteria [9]. Observational studies in adults with diabetes have identified a worsening of time to perform the timed up and go and five times sit-to-stand tests [4], walking speed [10], and greater strength deficit at high movement speeds [11]. Furthermore, another important point is the prediction in relation to physical performance tests. Low walking speed [12], performance on the Short Physical Performance Battery (SPPB) [13] and the Timed Up and Go (TUG) [14] tests, low muscle strength [15], and cardiorespiratory fitness [16], for example, have been associated with mortality.

Among the several factors involved in the relationship between diabetes and functional capacity, older adults with diabetes, in addition to presenting the common impairments of aging (i.e., neuromuscular, body composition, and metabolism changes), have added to this, complications and comorbidities resulting from the disease. Less is known about this relationship in middle-aged individuals, in which the impact of diabetic complications associated with the disease is also less known. However, exploratory evidence indicates that diabetes was associated, to a small extent, with physical disability in midlife [17]. Likewise, diabetes contributes to explaining the variance in the age trajectory of physical disability [18]. In this sense, socioeconomic and behavioral elements may be associated with the development and maintenance of diabetes. Results suggest a link between socioeconomic status and risk factors for type 2 diabetes, with an emphasis on sociodemographic factors, including age, ethnicity, family history, low education, and socioeconomic status, obesity, and unhealthy lifestyle behaviors (such as low levels of physical activity, sedentary time, and nutrient-poor diet) [19]. These effects are related throughout the entire life course. Furthermore, models of the physical disability process are longitudinal in nature and assume that interactions between the individual and their social, psychological, and physical environments are fundamental elements in the development of functional limitations throughout life [20, 21].

Individuals with diabetes are less likely to engage in regular physical exercise, even if this is one of the cornerstones of management [22]. Clinical trials such as the Look AHEAD Study [23] and Italian Diabetes and Exercise Study [24] demonstrated that physical activity interventions comprising lifestyle programs increased physical performance in patients with type 2 diabetes [23–26]. However, such findings are still inconsistent in other exercise trials [27, 28]. Such divergent results could be partly affected by several outcomes used in functional capacity and training specificity leading to variable degree of preparation for actual functional testing. In addition to the divergent results in primary studies, there is a strong focus on glycemic control in synthesis studies, and we have not identified a previous synthesis for functional capacity outcomes in this population.

Therefore, the purpose of this systematic review was to systematically assess the effect of different physical exercise modalities in patients with type 2 diabetes with an average age of 45 years or older on several outcomes used to measure functional capacity. Therefore, we conducted a preregistered protocol to summarize randomized controlled trials (RCTs) or non-randomized controlled studies (NRS) that assessed the changes (if any) of different modes of exercise training in outcomes related to the functional capacity of individuals with type 2 diabetes undertaking structured physical exercise compared with their non-training counterparts.

## Methods

This systematic review and meta-analysis was reported following the Preferred Reporting Items for Systematic Reviews and Meta-Analysis (PRISMA) guidelines [29] and our methodological approach followed the recommendations of the Cochrane Handbook for Systematic Reviews of Interventions, Version 6.1, 2020 [30].

The study was registered in the PROSPERO International prospective register of systematic reviews (registration number CRD42020162467) and followed the Preferred Reporting Items for Systematic Review and Meta-Analysis Protocols (PRISMA-P) [31]. The methodological protocol was uploaded to the Open Science Framework (OSF) (Preprint https://doi.org/10.31219/osf.io/kpg2m).

### Search Strategy

Potential studies were identified by using a systematic search process and were being conducted in the following databases: PubMed (via website), PEDro Physiotherapy Evidence Database (via website), Cochrane Library (via website), SPORTDiscus (via Periódicos CAPES), and Lilacs (via BVS). To minimize the prospect of publication bias, searches in Open Grey and Google Scholar were undertaken. The searches were carried out from inception until December 10, 2021.

The search strategies were developed using medical subject headings (MeSH) and EXPLODE TREES for terms: Aged, Exercise Therapy, Exercise Movement Techniques, Exercise, associated with synonyms for identification in title and summary (TIAB). Terms with study design different from clinical trials were used for identification in the title (TI) and exclusion. Search strategies can be found in Additional file 1 (Appendix 1).

### Study Selection

The review process was conducted by pairs of independent reviewers (eligibility process of titles and abstracts, full-text reading, and data extraction). Any disagreement in the study selection or extraction data processes was solved by consensus, referring back to the original articles or, if needed, by a third external reviewer (DU).

Six reviewers independently (LOP and LXNS, ATD and DMN, CEB and JLT) conducted a pilot of 400 articles, at the level of titles and abstracts, to standardize the eligibility criteria among the reviewers. These reviewers subsequently assessed titles and abstracts according to eligibility criteria using the EndNote bibliographic reference management software) and finally read the remaining full-text articles potentially eligible for inclusion.

Eligibility criteria were established based on the concept of population, intervention, comparator/control, outcome and study design (PICOS).

#### Type of Studies

We included randomized controlled trials (RCTs) or non-randomized controlled studies (NRS) published between January 1987 and January 2021. Although we did not restrict searches for specific languages, only articles in English, Spanish, or Portuguese were included.

#### Participants

Studies that included individuals (average age of 45 years or older, both sexes) with a diagnosis of type 2 diabetes, with or without comorbidities associated with the disease, were eligible for inclusion.

We excluded studies with patients who were diagnosed with neurodegenerative diseases (ataxias, Alzheimer's, Parkinson's); neuromuscular diseases (congenital/progressive, for example, dystrophies, myopathies), or musculoskeletal problems, such as fractures in general (hip, ankle, wrist, etc.) or any other injury that could interfere with the predicted functional tests; severe cognitive impairment (dementia, memory loss and confusion); severe cardiovascular disease (congestive heart failure) or recent cardiovascular events (within the last 6 months, such as acute myocardial infarction or stroke); and cancer in the treatment period.

#### Type of Interventions

We included all trials which reported the interventions with structured physical exercise (e.g., resistance training, power training, aerobic training or combined training; pilates, functional training, etc.) lasting at least eight weeks. We considered purely structured exercise interventions. Studies were discarded if they presented another co-intervention with physical exercise, for example, diet, food supplements, health education, or behavior change/lifestyle interventions.

The comparator could not practice any type of physical activity/exercise component, nor could they participate routinely during the period of study of groups with exercise guidance or lifestyle changes.

#### Outcome Measures

To account for measures of functional capacity more comprehensively, any of the following outcomes were considered for inclusion:

1. Timed Up and Go test (TUG);
2. Chair stands (5-chair stand test; 30-s chair stand test);
3. Walking performance (6-min walk, 400-m walk);
4. Upper-limb muscle strength evaluated by strength isometric (handgrip);
5. Lower-limb muscle strength assessed by the test of one repetition maximum (1RM), (knee extension or leg-press);
6. Physical fitness parameter evaluated by maximal oxygen consumption (VO_2max_) or peak oxygen consumption (VO_2peak_).

### Data Extraction

The six reviewers mentioned above (LOP, LXNS, ATD, DMN, CEB and JLT) performed data extraction in a sheet that was designed and tested before use. The information from the eligible studies was coded and grouped into four categories: (1) general study descriptors (authors, year of publication, journal, study design); (2) description of the study population (e.g., sex, age, total sample size, health-related data); (3) details of interventions (e.g., type, duration, frequency, intensity); (4) and outcomes (e.g., functional parameters, walking performance, muscle strength parameters, physical fitness parameters). For continuous outcomes, we extracted the results with raw data of means and standard deviations (SDs) and delta values when available.

When data were not available, we contacted the corresponding author(s) to request the missing data. It was not necessary to input any data. We only calculated, in some cases, the delta to observe the difference between the pre- and post-intervention moments of the outcomes of interest.

#### Quality Assessment and of the Risk of Bias in Individual Studies

Paired reviewers independently evaluated the risk of bias for each selected study using the Downs & Black checklist [32], which allows the assessment of both randomized and non-randomized trials, in regard to the following items: reporting, external validity, internal validity (bias), internal validity (confounding—selection bias), and power. To determine the methodological quality and risk of bias of a study, for each criterion, we evaluated the presence of sufficient information. Disparities were resolved by involving a third author. The last item on the checklist (power of analysis) was used in a binary approach with a score of “0” (no sample size calculation) or “1” (reported sample size calculation) [33]. The checklist is composed of 27 questions, with a total possible score of 28 for randomized and 25 for non-randomized studies, and the following scoring ranges: excellent (26–28); good (20–25); fair (15–19); and poor (≤ 14).

### Data Synthesis

Meta-analyses and the forest plots were performed in R version 4.0.1 (R Project for Statistical Computing, RRID:SCR_001905), using the metafor package, for the outcomes of interest that presented at least two studies and/or group combinations.

We used the inverse-variance method (DL − tau^2^), under a random-effects model, to generate effect estimates. Because our results are derived from continuous outcomes with the same scale available, we used the mean difference with 95% confidence intervals (95% CI) [30]. We also calculated the prediction interval when at least three studies were available in a given meta-analysis [34]. The evaluation of heterogeneity across trials was assessed by generating the I^2^ statistic, which represents the proportion of heterogeneity that is not due to chance (rather, due to possible differences across studies, populations, and interventions).

#### Additional Analyses

As planned in our study protocol [35], when sufficient data (at least 10 studies) were available, we performed sex-stratified subgroup analysis and meta-regression with glycated hemoglobin (HbA1c) values. We also conducted a sensitivity analysis stratifying for randomized or non-randomized studies. Regarding the duration of diabetes diagnosis, we split study samples by short- and long-term duration of the disease (> 8 years). In addition, we used the “leave-one-out” approach to check whether removing a single study at each time has had a major influence (e.g., change in the direction of results) on meta-analytic estimates. The publication bias was assessed by visual inspection through the generation of a funnel plot.

It was not possible to carry out a sensitivity analysis, as we had planned, with patients with neuropathy, as none of the studies reported a population with this comorbidity.

## Results

### Description of Included Studies

**Fig. 1:**
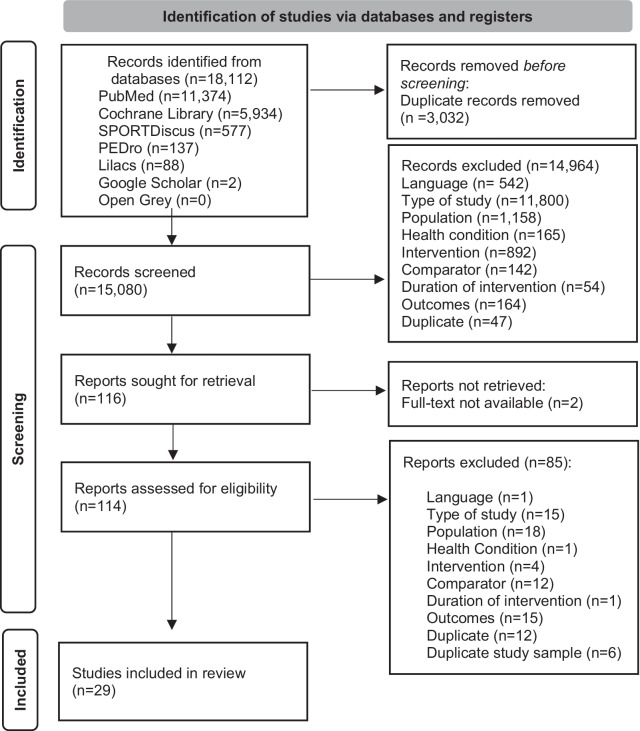
PRISMA flow diagram

From 18,112 articles retrieved from the electronic database, 14,964 were excluded by titles and abstracts. Out of 116 reviewed full-texts, 25 RCTs [36–60] and 4 NRS [61–64] met the inclusion criteria (Fig. 1), representing a total sample of 1,557 participants. Of these, 489 patients were included in studies of aerobic exercise training, 193 in studies of resistance exercise training, 386 in combined aerobic/resistance exercise training studies, 375 in studies with two or more intervention arms (aerobic/combined or aerobic/resistance/combined), and 114 in others (i.e., Pilates, Tai Chi, Whole-body vibration). The articles were mostly published in English, except for 1 article in Portuguese.

In addition, we cite some studies that might appear to meet the inclusion criteria but were excluded due to the control group [65, 66] (received thematic sessions with topics on nutrition and physical activity, for example, participated in a 12-session health promotion educational training), an apparently duplicated sample with included study [67], and because of the intervention (diet plus supervised exercise) [68].

Overall, the median age from participants’ samples was 60 (minimum and maximum: 52–73) years old. No studies included participants with peripheral neuropathy. Regarding the sexes of participants enrolled in the included studies, 20 study samples consisted of both women and men, six studies included only men, whereas three studies included only women (Table 1).

**Table 1:** Characteristics of the studies included

Authors	Control group intervention	Design	Outcomes	Sample size	Other clinical conditions	Baseline HbA1c (%), Mean (SD)	Duration of the disease (y), range or mean (SD)	Medications	Sex, female (%)	Age (y), mean (SD)
Jiang et al. [47]	Required to maintain their usual physical activity	RCT	Body composition FATmax VO_2max_ Blood chemistry Physical capacity	49	Postmenopausal	6.72(0.7)	6 to 11 (range)	Metformin Sulfonylureas ACE inhibitors Diuretics Statins Fibrates	49	63(5)
Yamamoto et al. [37]	Instructed to maintain their daily activities	RCT	Muscle strength Gait speed Body composition	53	NR	7.24(0.77)	17.0 (10.3)	NR	47	73(2)
Shabkhiz et al. [36]	Instructed to maintain their normal activities and not to modify their lifestyles	RCT	Blood chemistry Muscle strength Body composition	44	NR	NA	10.2(3)	Insulin-secretagogue Insulin-sensitizer Lipid lowering Anti-hypertensive	0	72(6)
Hwang et al. [39]	Instructed not to change their habitual physical activity, diet, or medications	RCT	VO_2peak_ Body composition Blood chemistry Habitual physical activity Dietary analysis	50	NR	7.23(0.33)	8(1)	Metformin SGLT2 inhibitors Sulfonylureas DPP-4 inhibitors GLP-1 agonists ThiazolidinedionesInsulin Statins Anti-hypertensives Aspirin	46	63(1)
Wilson et Al. [60]	Instructed to maintain their usual lifestyle	RCT	VO_2peak_ Left ventricular function Body composition Blood volume	16	NR	7.77(3.61)	7.2(4.2)	Metformin Gliclazide Insulin	37.5	52(8)
Scheer et al. [61]	Instructed to maintain their usual activities	NRS	VO_2peak_ Anthropometric variables Blood chemistry Muscular strength Vascular function	27	Obese Overweight	7.1(0.84)	NR	Biguanides Sulfonylureas GLP-1 agonists DPP-4 inhibitors Statins Beta blockers Calcium channel blockers ACE inhibitors Angiotensin II receptor antagonist Anti-inflammatories Diuretic Fibrate Thyroid hormones Estrogen Testosterone, Paracetamol Other pain relief	44	62(10)
Conners et al. [38]	Instructed to maintain their current dietary and physical activity habits	RCT	Glycemic control Blood lipids Health-related fitness	26	NR	7.58(NR)	7.1(4.6)	Metformin Sitagliptin	61	58(5)
Szilágyi et al. [40]	Did not participate in any exercise	RCT	Plasma glucose Body composition Physical fitness level	208	NR	NA	20.4(7)	NR	64	61(7)
Melo et al. [41]	Received guidance for maintenance of medication and the nutritional intake of foods consumed in the diet	RCT	Plasma glucose HbA1c Functional capacity	22	NR	7.6(0.75)	8.3(6)	Metformin Glibenclamide Sitagliptin Glimepiride	100	67(7)
Banitalebi et al. [59]	Usual medical care and received diabetes recommendations for self-management. Were not given exercise counselling and were asked to maintain physical activity levels	RCT	Myokine levels Metabolic outcomes Body composition VO_2peak_	42	Overweight	9.41(0.82)	NR	NR	100	55(6)
Santos et al. [62]	Received no intervention and were instructed not to change their lifestyle	NRS	Maximal strength	48	NR	NA	NR	Hypoglycemic agents	63	67(5)
Pozo-Cruz et al. [42]	Receiving only standard care	RCT	Glycemic control Dyslipidemia Functional capacity	39	NR	7.17(0.96)	9.2(7.7)	NR	49	69(10)
Yan et al. [58]	–	RCT	Blood pressure Body composition Blood chemistry VO_2max_	41	Hypertension	8.7(2.8)	NR	Nifedipine Amiloride Hydrochlorothiazide Methyldopa Enalapril Atenolol Chlorthalidone Metformin Glyburide	0	53(11)
Tan et al. [43]	Instructed to maintain their individual habits of physical activities and refrain from engaging in any other forms of prescribed exercise training	RCT	Body composition Glycemic control Lipid profile Functional capacity	25	NR	6.38(0.97)	16.7(6.7)	Oral hypoglycemic	48	66(4)
Labrunée et al. [48]	Received counsels regarding physical activity practice	RCT	Anthropometric variables Blood chemistry Physical capacities Maximal isometric strength QOL	23	Obesity (stage 2–3)	8.67(1.81)	> 1 year	Insulin Metformin Sulfonylureas	56.5	53(9)
Karstoft et al. [52]	Were instructed to continue their habitual lifestyle	RCT	VO_2max_ Body composition Blood pressure Blood chemistry	32	NR	6.66(0.2)	4.7(1.2)	Metformin Sulfonylureas DPP-4 inhibitors GLP-1 analogues	31.57	59(2)
Kadoglou et al. [54]	Maintenance of usual activities	RCT	VO_2peak_ Body composition Blood chemistry	89	Overweight or Obese	8.02(1.04)	6.3(3.3)	Metformin Gliclazide	63	59(8)
Plotnikoff et al. [57]	Non-training and maintenance of physical activity levels	RCT	Muscle strength Blood chemistry Body composition Social cognitions	48	Obese	6.86(1.21)	NR	Insulin Metformin Sulfonylureas Thiazolidinediones α-glucosidase inhibitors ACE inhibitors Angiotensin receptor blockers Diuretics β-blockers Calcium channel blockers Statins Fibrates Cholesterol absorption inhibitors Aspirin	67	55(12)
Balducci et al. [44]	Remained sedentary	RCT	Biochemical parameters VO_2max_ Body composition Volume of physical activity	82	Metabolic syndrome	7.41(1.41)	8.9(6)	Sulfonylurea Glinide Metformin Thiazolidinedione Insulin ACE inhibitors Angiotensin-receptor blocker Diuretic Calcium-channel blocker β-blocker α1-adrenergic blocker Statins Fibrates Antiplatelet agents	40.32	62(8)
Larose et al. [50]	Instructed to revert to their level of activity at baseline and to maintain this level	RCT	VO_2peak_ Submaximal exercise response Muscular strength	251	Obesity	7.68(0.88)	5.3(4.4)	NR	36.2	54(7)
Loimaala et al. [55]	Standard treatment for type 2 diabetes	RCT	Cardiovascular risk factors Arterial pulse wave velocity Blood chemistry Muscle strength VO_2max_	48	Hypertension	8.1(1.2)	NR	Metformin Sulfonylureas	0	54(6)
Lam et al. [45]	Wait list control	RCT	Blood chemistry Blood pressure Body composition Health status Functional capacity	53	NR	8.54(1.25)	NR	Insulin	54.71	62(10)
Brun et al. [49]	Usual routine treatment	RCT	Lifestyle and fitness outcomes Body composition Metabolic outcomes QOL Healthcare costs	25	Overweight Obesity	8.86(1.35)	10(7)	NR	26	60(10)
Kadoglou et al. [53]	Maintenance of usual activities	RCT	Body composition VO_2peak_ Blood chemistry Blood pressure	60	Overweight	7.88(0.96)	6.8(4.1)	Sulfonylurea Metformin Antihypertensives	57	62(5)
Bjørgaas et al. [46]	Not given any specific recommendations concerning physical activity	RCT	VO_2max_ Fitness, clinical and laboratory variables	29	Overweight	7.4(1.2)	NR	Metformin Sulfonylurea Antihypertensives Lipids-lowering Aspirin	0	57(8)
Fritz et al. [63]	Received no exercise instructions	NRS	Blood chemistry Blood pressure Body composition VO_2max_	52	NR	6.15(0.8)	5.5(4.3)	Glucose lowering agents Antihypertensives Lipids-lowering	50	60(7)
Loimaala et al. [51]	Received conventional treatment of type 2 diabetes only	RCT	Body composition Blood chemistry VO_2max_ Muscle endurance Isometric strength Baroreflex sensitivity Heart rate variability Whole-body impedance cardiography	49	Hypertension	8.1(1.69)	> 3 years	Hypoglycemic agents	0	53(5)
Verity et al. [56]	Instructed to maintain their normal daily activities	RCT	Body composition Blood chemistry VO_2max_	10	Postmenopausal Overweight	8.85(1.79)	4.5	None	100	59(12)
Skarfors et al. [64]	Not physical training	NRS	VO_2max_ Blood chemistry	16	Musculoskeletal problems Asthma on exertion Hypertension only control group	NA	2.6(3)	Digoxin Antihypertensives Sulfonylurea Bronchodilators	0	59(2)

*SD* Standard deviation; *RCT* randomized controlled trial; *NRS* non-randomzsed controlled Study; *NR* not reported; *NA* not applicable; *VO*_*2max*_ maximum oxygen volume; *VO*_*2peak*_ peak oxygen consumption; *QOL* quality of life; *ACE* angiotensin-converting enzyme inhibitor; *DPP-4* dipeptidyl peptidase-4 inhibitors; *SGLT2* sodium-glucose cotransporter-2 inhibitors

### Intervention Characteristics

Among the 29 studies included, 13 studies used aerobic training [38, 39, 47–49, 52–54, 56, 58, 60, 63, 64], six used combined training (aerobic and resistance) [40, 43, 46, 51, 55, 61], four studies used resistance training [36, 37, 57, 62], three studies used more intervention arms [44, 50, 59] (two studies with aerobic training groups and combined training, and one with aerobic, resistance and combined training groups) and three studies with another type of training (Pilates, Tai Chi, Whole-body vibration) [41, 42, 45] (Table 2).

**Table 2:** Characteristics of studies’ interventions

Authors	Intervention setup	Frequency, times per week	Intensity, range or mean (SD)	Time for intervention, minutes per session, range	Average length, weeks
Jiang et al. [47]	Aerobic	3	41.3(3.2) to 46.1(10.3)% VO_2max_	20 to 60	16
Yamamoto et al. [37]	Resistance	7	1.3 to 3.3 kg	NR	48
Shabkhiz et al. [36]	Resistance	3	70% 1RM	NR	12
Hwang et al. [39]	Aerobic	4	70 to 90% HR_peak_	40 to 47	8
Wilson et al. [60]	Aerobic	3	90% HR_peak_	20	13
Scheer et al. [61]	Combined	3	60 to 80% HR_max_; 12 to 15 RPE Borg Scale	60	8
Conners et al. [38]	Aerobic	3	40 to 70% HRR	10 to 20	12
Szilágyi et al. [40]	Combined	4	60 to 75% Max. pulse	60	24
Melo et al. [41]	Pilates	3	11(1) to 12(1) RPE Borg Scale	60	12
Banitalebi et al. [59]	Aerobic, Combined	3	10 to 15 RM; 50 to 70% HR_max_	50	10
Santos et al. [62]	Resistance	3	50 to 70% 1RM	50	16
Pozo-Cruz et al. [42]	Whole-body vibration	3	12 to 16 Hz	8 to 16	12
Yan et al. [58]	Aerobic	3 to 5	50 to 75% VO_2peak_	45	12
Tan et al. [43]	Combined	3	55 to 70% HR_max_ 50 to 70% 1RM	60	26
Labrunée et al. [48]	Aerobic	7	HR% (the first ventilatory threshold measured the test of effort)	30	13
Karstoft et al. [52]	Aerobic	5	55 to 70% peak energy-expenditure rate	60	17
Kadoglou et al. [54]	Aerobic	4	50 to 80% VO_2peak_	45 to 60	52
Plotnikoff et al. [57]	Resistance	3	50 to 85% 1RM	NR	16
Balducci et al. [44]	Aerobic, Combined	2	70 to 80% VO_2max_; 80% 1RM	60	52
Larose et al. [50]	Aerobic, Resistance, Combined	2 to 3	60 to 75% HR_max_; 8 to 15 RM	20 to 45	22
Loimaala et al. [55]	Combined	4	65 to 75% VO_2max_; 60 to 80 MVC	30	104
Lam et al. [45]	Tai Chi	1 to 2	NR	60	26
Brun et al. [49]	Aerobic	2	HR% (level of the ventilatory threshold)	45	52
Kadoglou et al. [53]	Aerobic	4	50 to 75% VO_2peak_	45 to 60	26
Bjørgaas et al. [46]	Combined	2	50 to 85% HR_max_	90	12
Fritz et al. [63]	Aerobic	3	NR	45	17
Loimaala et al. [51]	Combined	2	65 to 75% VO_2max_; 70 to 80% 1RM	≥ 30	52
Verity et al. [56]	Aerobic	3	65 to 80% HRR	60 to 90	16
Skarfors et al. [64]	Aerobic	3	Up to 75% VO_2max_	45	104

*NR* not reported; *VO*_*2max*_ maximum oxygen volume; *VO*_*2peak*_ peak oxygen consumption; *HR*_*max*_ maximum heart rate; *HRR* heart rate reserve; *HR* heart rate; *HR*_*peak*_ peak heart rate; *Max. pulse* maximum pulse; *1RM* one maximum repetition; *RM* maximum repetition; *MVC* maximal voluntary contraction; *kg* kilogram; *Hz* hertz; *RPE* rating of perceived exertion

The mean training duration was 27.9 weeks (range: 8 to 104 weeks). Training frequency ranged from one to seven days per week, with three days a week the most employed training frequency (*n* = 14). The exercise sessions duration ranged from 8 to 90 min/exercise/session.

In aerobic training, the most used measures were maximal oxygen uptake (VO_2max_), peak oxygen uptake (VO_2peak_), maximum heart rate (HR_max_), and heart rate reserve (HRR), and for those of resistance training were one repetition maximum (1RM) and repetitions maximum (RM). In studies that used HRmax or peak heart rate (HR_peak_) to quantify aerobic exercise intensity, programs ranged from 50 to 90% intensity, whereas they ranged from 40 to 80% when HRR was used as an intensity variable. VO_2peak_ ranged from 50 to 90% VO_2peak_; VO_2max_ ranged from 65 to 80% VO_2max_. 1RM ranged from 50 to 80% 1RM and RM ranged from 8 to 15 RM.

The intensity measures less commonly used in the studies were: heart rate (HR%); peak energy-expenditure rate (55 to 70%); maximum pulse (60 to 75%); rating of perceived exertion (RPE) (12 to 15/11(1) to 12(1) RPE Borg Scale); maximum voluntary contraction (MVC) (60 to 80 MVC); 1.3 to 3.3 kg; 12 to 16 Hz. Only two studies did not report intensity of interventions.

### Functional Capacity

Among the outcomes prespecified in the study protocol, the 400-m walk test was not assessed in the included studies. The results of the remaining outcomes of interest are presented below.

#### Walking Performance

**Fig. 2:**
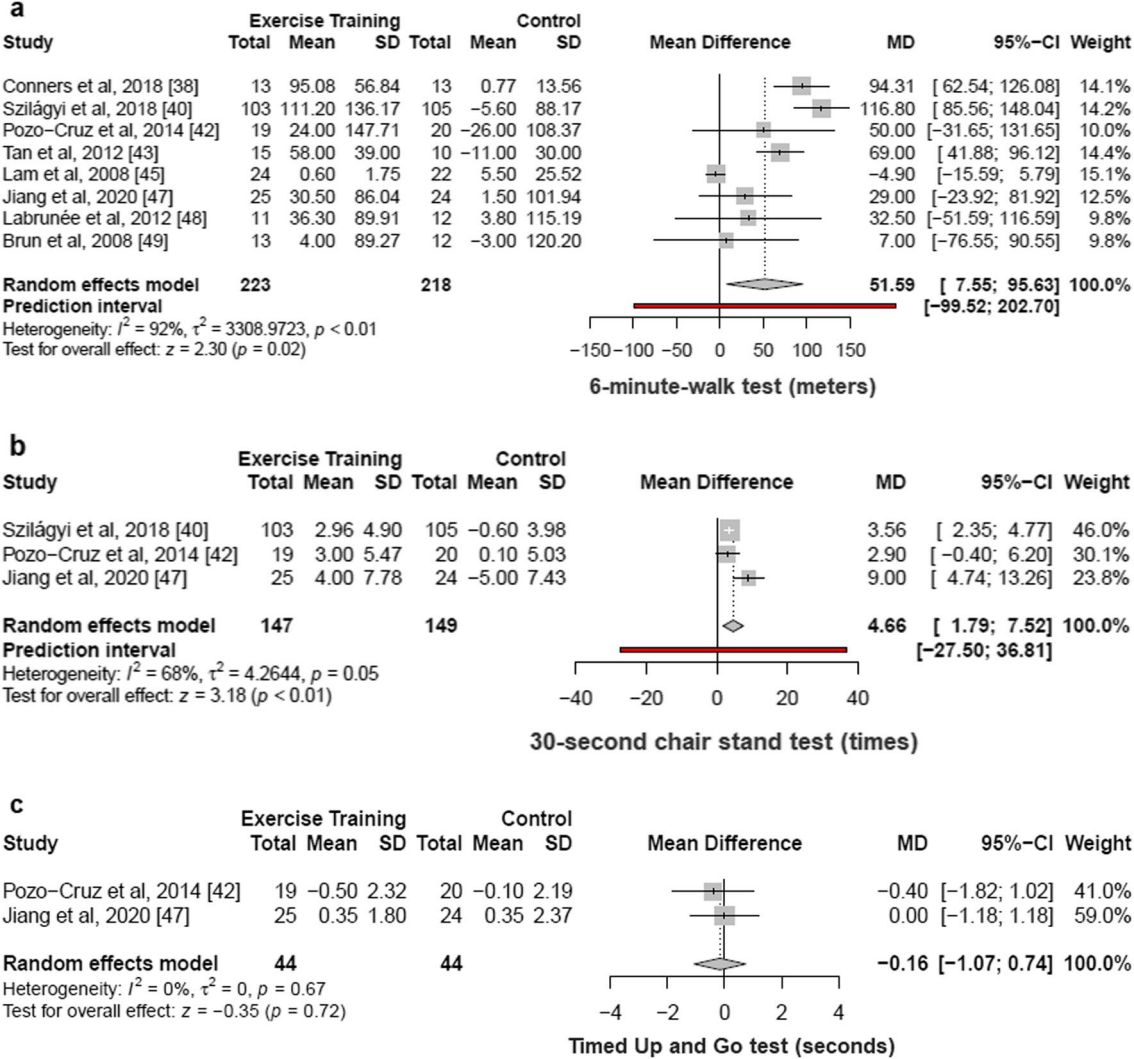
Functional capacity outcomes. Meta-analysis of included studies comparing changes in walking performance (**a**), chair stands (**b**), and timed up and go test (**c**) by structured physical exercise *vs* control. CI indicates confidence interval. Changes in 6-min walk test, 30-s chair stand test, and timed up and go test of individual studies included in the meta-analysis of structured physical exercise *vs* no intervention in patients with type 2 diabetes

Out of the 29 included studies, eight articles [38, 40, 42, 43, 45, 47–49] with 441 patients demonstrated that structured physical exercise interventions were associated with an increase of 51.59 m in walking performance evaluated by the 6-min walk test (6MWT) (95% CI 7.55% to 95.63%; I^2^ 92%; *p* for heterogeneity < 0.01) as compared with control (Fig. 2a).

#### Chair Stands

Three articles (296 patients) [40, 42, 47] demonstrated that structured physical exercise interventions were associated with an increase of 4.66 times in 30-s chair stand test (95% CI 1.79% to 7.52%; I^2^ 68%; *p* for heterogeneity = 0.05) as compared with control (Fig. 2b).

One study reported the 5-chair support test [41], and there were significant improvements for the Pilates intervention group compared with the control (Δ mean: intervention group -4 s; control group 1.3 s).

#### Timed Up and Go Test

Two articles (88 patients) [42, 47] demonstrated that structured physical exercise interventions were associated with a decrease of 0.16 s in the performance of the timed up and go test (95% CI − 1.07% to 0.74%; I^2^ 0%; *p* for heterogeneity = 0.67) as compared with controls (Fig. 2c).

#### Lower-Limb Muscle Strength

**Fig. 3:**
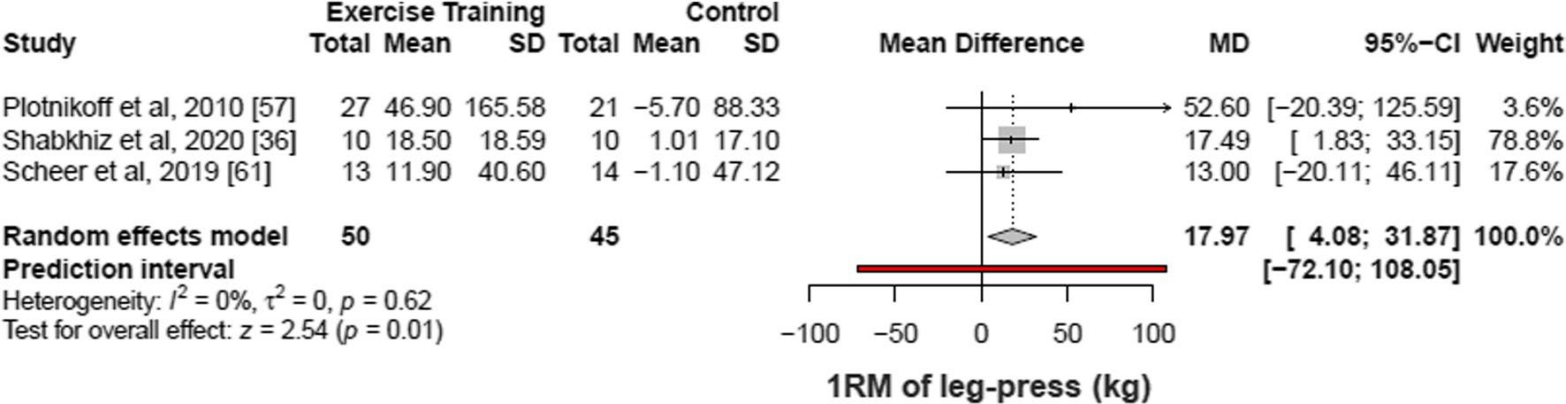
Meta-analysis of included studies comparing changes in one repetition maximum by structured physical exercise *vs* control. CI indicates confidence interval. Changes in the strength of lower-limb muscle evaluated by 1RM of leg-press test of individual studies included in the meta-analysis of structured physical exercise *vs* no intervention in patients with type 2 diabetes

Out of the 29 included studies, three articles (95 patients) [36, 57, 61] demonstrated that structured physical exercise interventions were associated with an increase of 17.97 kg in the strength measures of lower-limb muscle evaluated by 1RM of leg-press (95% CI 4.08% to 31.87%; I^2^ 0%; *p* for heterogeneity = 0.62) as compared with control (Fig. 3). Another study [62] showed an increase in muscle strength evaluated by the 1RM of knee extension test for the intervention group in relation to control [62] (Δ mean: intervention group 5.03; control group 0.8).

#### Upper-Limb Muscle Strength

One study [37] reported isometric strength assessed by handgrip and showed no differences (Δ mean: intervention group 0.3; control group − 0.03).

#### Physical Fitness

**Fig. 4:**
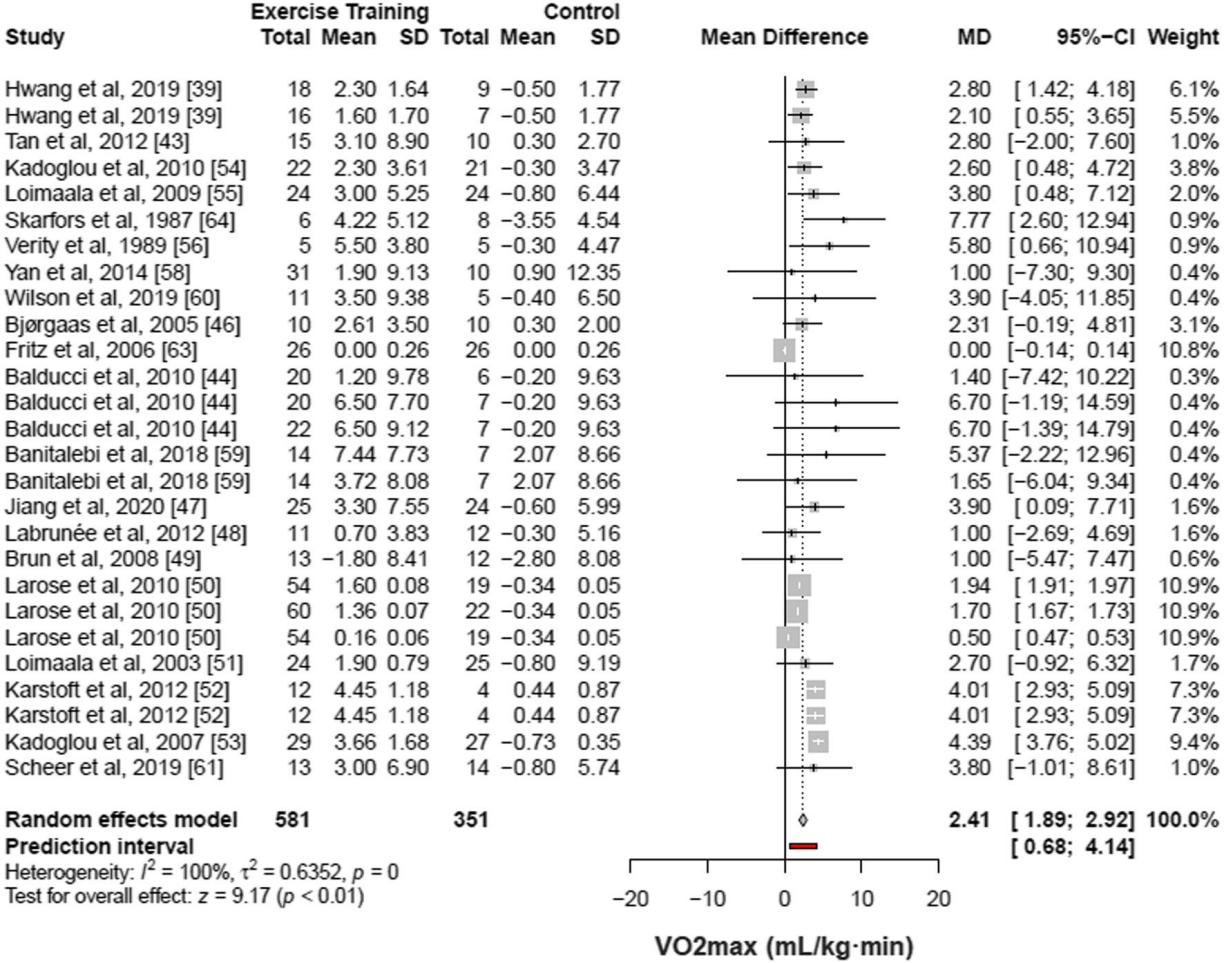
Meta-analysis of included studies comparing changes in maximal oxygen consumption by structured physical exercise *vs* control. CI indicates confidence interval. Changes in physical fitness evaluated by VO_2max_ of individual studies included in the meta-analysis of structured physical exercise *vs* no intervention in patients with type 2 diabetes. Studies that included more than 1 modality or different training protocols within the same type of structured physical exercise were evaluated as separate observations

Out of the 29 included studies, 20 articles [39, 43, 44, 46–56, 58–61, 63, 64] with 27 groups of comparison (932 patients) demonstrated that structured physical exercise interventions were associated with an increase of 2.41 mL/kg·min in VO_2max_ (95% CI 1.89% to 2.92%; I^2^ 100%; *p* for heterogeneity = 0) as compared with control (Fig. 4).

Of these, 12 studies [43, 44, 46, 47, 49, 51, 52, 55, 56, 58, 63, 64] presented the results of oxygen consumption in VO_2max_, being 10 studies [43, 44, 46, 47, 49, 51, 52, 55, 56, 58] with the unit of measure in mL/kg·min, one study [64] in mL/min and another study in L/min [63]. The last two studies were transformed to mL/kg·min using the body weight presented by each of the studies. The other eight studies [39, 48, 50, 53, 54, 59–61] had the measure of oxygen consumption in VO_2peak_ and all of them with the unit of measure in mL/kg·min. The results of VO_2max_ and VO_2peak_ were combined in the same meta-analysis.

### Additional Analyses

**Fig. 5:**
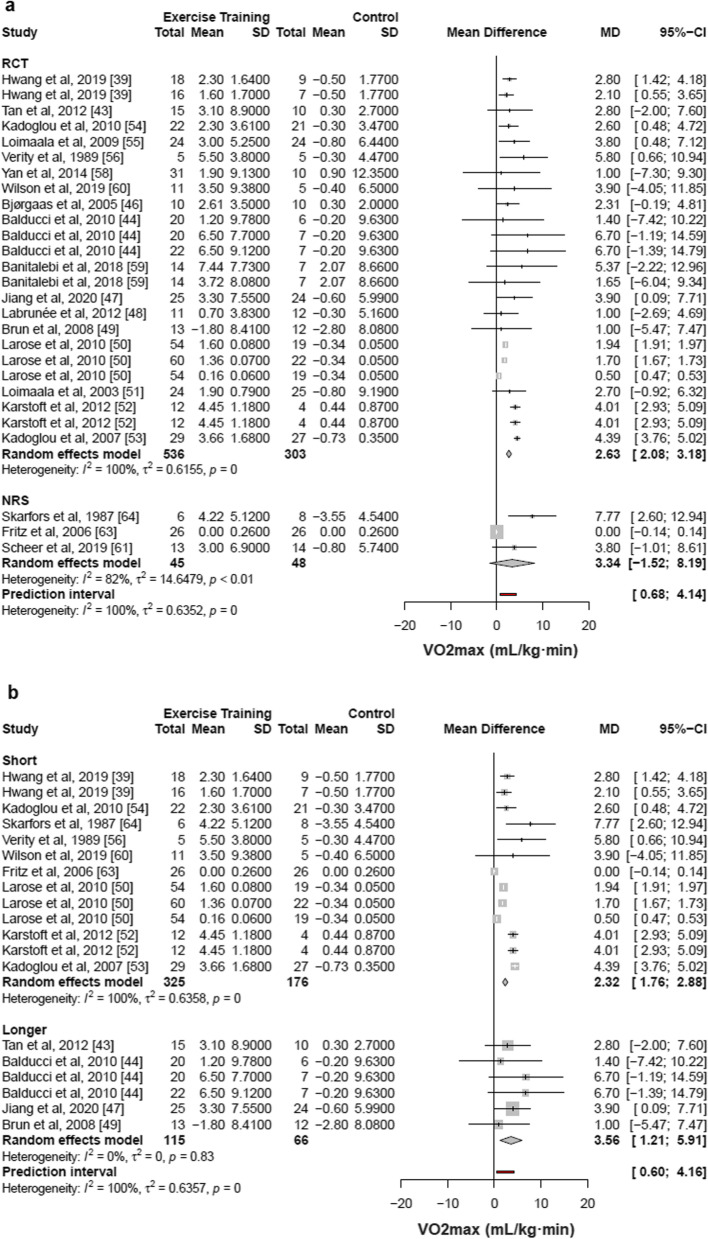
Sensitivity analysis for the type of study (**a**) and duration of diabetes diagnosis (**b**). CI indicates confidence interval. Changes in physical fitness evaluated by VO2max of individual studies included in the meta-analysis of structured physical exercise *vs* no intervention in patients with type 2 diabetes. Studies that included more than 1 modality or different training protocols within the same type of structured physical exercise were evaluated as separate observations. Structured physical exercise and control group in the randomized clinical trials (RCT) and non-randomized controlled studies (NRS). Structured physical exercise and control group with studies showing short and longer (> 8 years of diabetes) duration of type 2 diabetes

In sensitivity analysis, RCT studies [39, 43, 44, 46–56, 58–60] (17 studies, 24 comparisons, 839 patients) were associated with an increment of 2.63 mL/kg·min in the VO_2max_ (95% CI 2.08 to 3.18; I^2^ 100%, *p* for heterogeneity = 0) as compared with control. The NRS studies [61, 63, 64] (3 studies, 93 patients) were associated with an increment of 3.34 mL/kg·min in the VO_2max_ (95% CI − 1.52 to 8.19; I^2^ 82%, *p* for heterogeneity < 0.01) as compared with control (Fig. 5a). Regarding the duration of diabetes, we split study samples by short- and long-term duration of the disease (> 8 years). The studies that included diabetes of short duration [39, 50, 52–54, 56, 60, 63, 64] (9 studies, 13 comparisons, 501 patients) were associated with an increment of 2.32 mL/kg·min in the VO_2max_ (95% CI 1.76 to 2.88; I^2^ 100%, *p* for heterogeneity = 0) as compared to control. Studies that included diabetes with longer duration [43, 44, 47, 49] (4 studies, 6 comparisons, 181 patients) were associated with an increment of 3.56 mL/kg·min in the VO_2max_ (95% CI 1.21 to 5.91; I^2^ 0%, *p* for heterogeneity = 0.83) as compared to control (Fig. 5b).

When studies were individually omitted from the meta-analysis, heterogeneity was unchanged. A table with the values of the heterogeneity from each study can be found in Additional file 1 (Appendix 2).

**Fig. 6:**
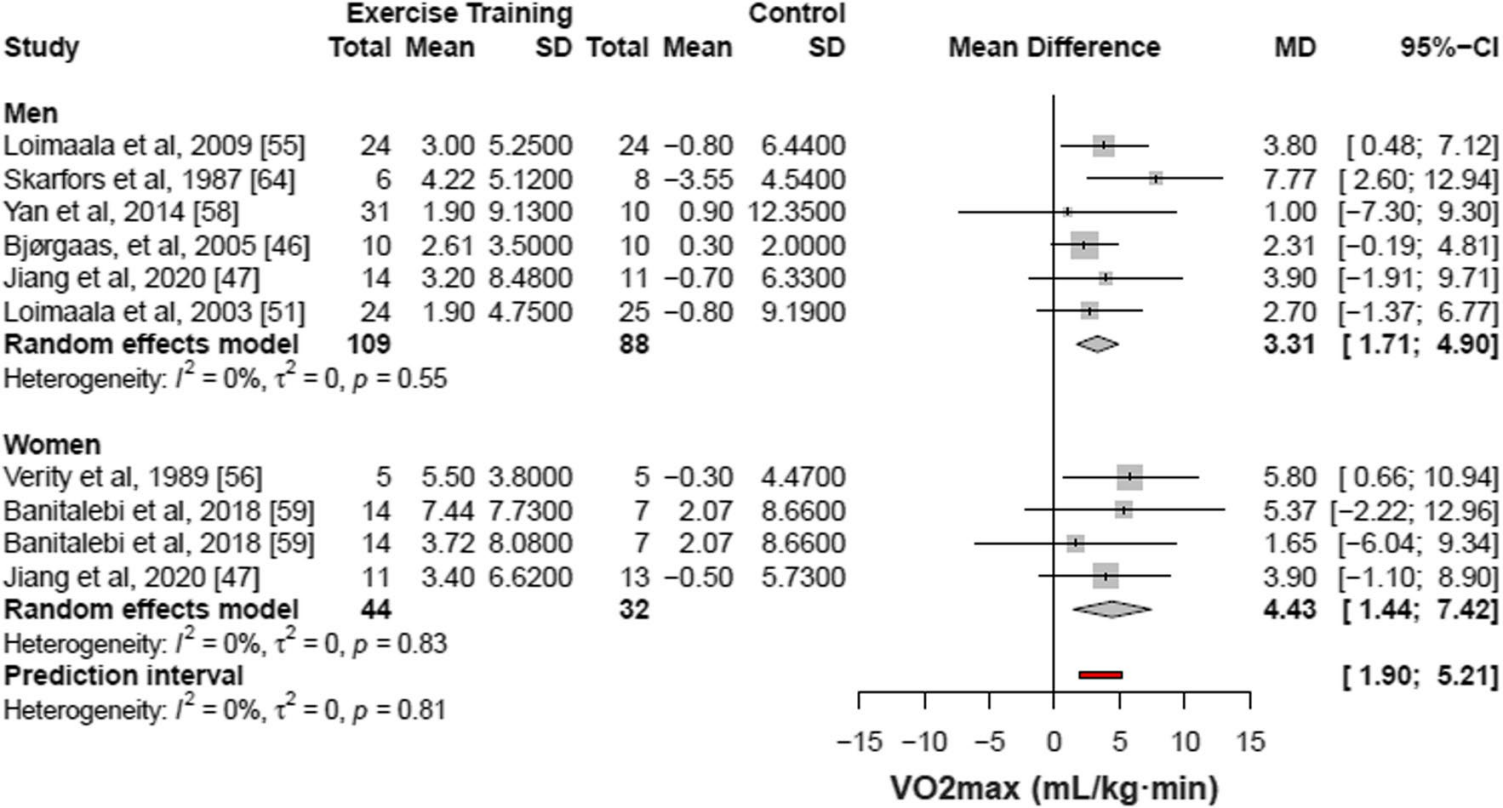
Subgroup analysis stratified by sex. CI indicates confidence interval. Changes in physical fitness evaluated by VO_2max_ of individual studies included in the meta-analysis of structured physical exercise *vs* no intervention in patients with type 2 diabetes. Studies that included more than 1 modality or different training protocols within the same type of structured physical exercise were evaluated as separate observations

In the subgroup analysis (Fig. 6), studies with women [47, 56, 59] (3 studies, 4 comparisons, 76 patients) showed that interventions were associated with an increase of 4.43 mL/kg·min in VO_2max_ (95% CI 1.44 to 7.42; I^2^ 0%, *p* for heterogeneity = 0.83) and studies with men [46, 47, 51, 55, 58, 64] (6 studies, 197 patients) showed that interventions were associated with an increase of 3.31 mL/kg·min in VO_2max_ (95% CI 1.71 to 4.90; I^2^ 0%, *p* for heterogeneity = 0.55), compared to control.

Meta-regression showed no association between HbA1c levels and VO_2max_ (*p* = 0.34; I^2^ 99.6%; *R*^2^ = 2.6%; *p* for heterogeneity < 0.0001). Publication bias was assessed using a contour-enhanced funnel plot of each trial’s effect size against the standard error. We did not find any publication bias (*p* = 0.76), and the funnel plot is presented in Additional file 1 (Appendix 3).

### Quality Assessment and Risk of Bias in Individual Studies

**Fig. 7:**
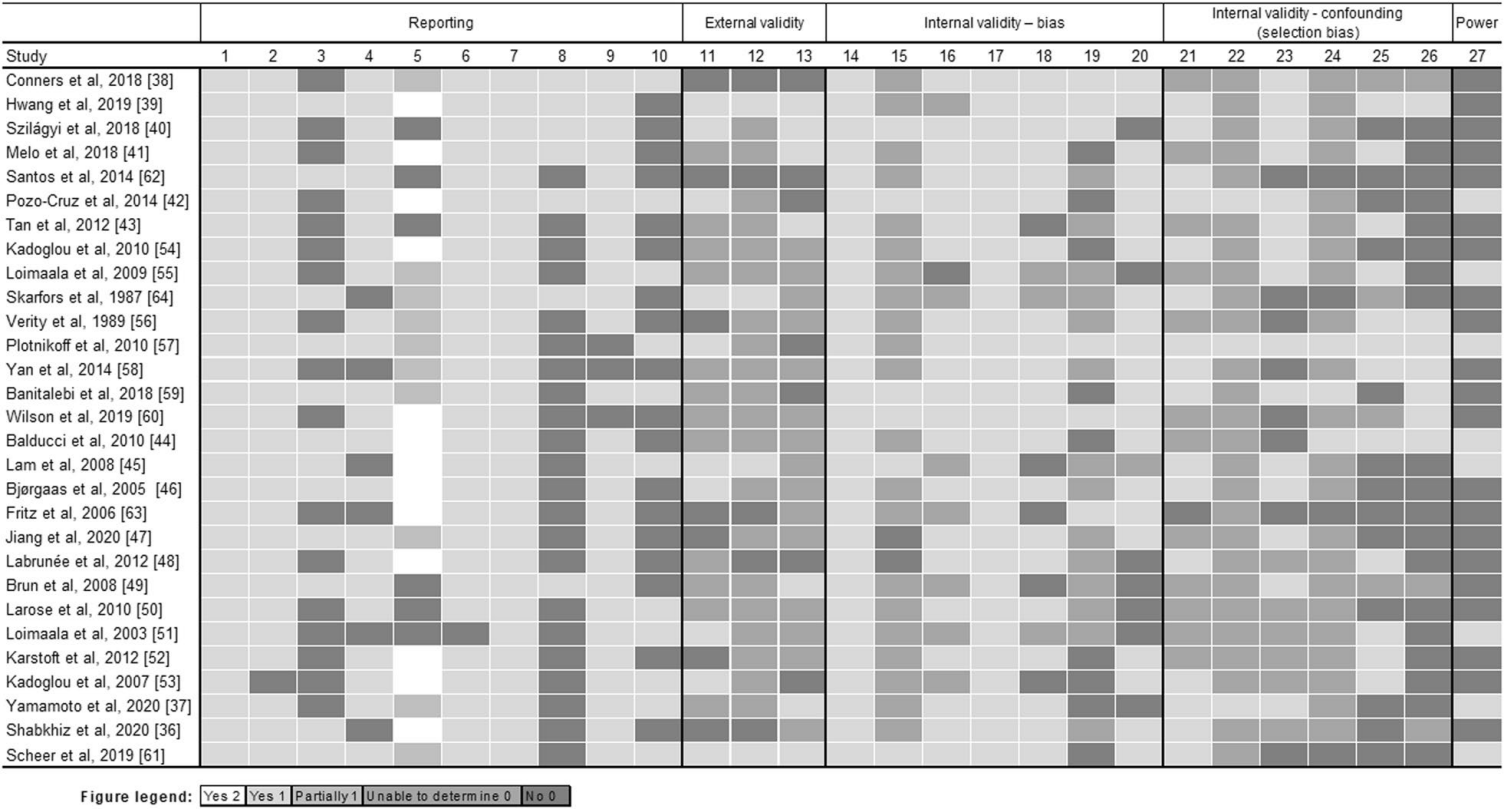
Risk of bias rating based on the Downs & Black checklist. Description: score for each item with their respective colors

The following items were evaluated with respect to reporting, external validity, internal validity (bias), internal validity (confusion—selection bias), and power. For item 14, we answered yes to all of the studies, because these are studies with exercise interventions, so the blinding of the participants generally does not occur. As noted previously, the checklist consists of 27 questions, with RCTs scoring up to 28 and NRS at most 25. Four studies [39, 42, 57, 61] scored good (20–25), 10 studies [37, 38, 40, 41, 44–46, 54, 59, 60] fair (15–19) and 15 studies [36, 43, 47–53, 55, 56, 58, 62–64] poor (≤ 14), with available data in Additional file 1 (Appendix 4). In Fig. 7, we represent the evaluation of the studies for each of the items present in the Downs & Black checklist [32].

## Discussion

This systematic review with meta-analysis summarizes the effects of exercise training on functional outcomes of people with type 2 diabetes. Although several syntheses have addressed exercise for patients with type 2 diabetes, the present study used a comprehensive assessment by including different functional outcomes. We observed in the current systematic review and meta-analysis that structured exercise programs might improve functional capacity as indicated by walking performance, chair stands, time up and go tests, 1RM of leg-press, and VO_2max_ in people with type 2 diabetes. In additional sensitivity and meta-regression analyses, we could not identify isolated factors or studies that may had a differential influence on summary estimates. Most studies’ scores indicate a high risk of bias, which underscores the importance of careful interpretation regarding the summarized evidence. Most of the studies included participants with an average age close to 60 years or more; therefore, our results are more widely generalizable to patients with type 2 diabetes over 45 years old.

The present meta-analysis demonstrated that cardiorespiratory fitness, measured by VO_2max_, can be improved with structured physical exercise interventions in people with type 2 diabetes, supporting previous observations in this population [69, 70]. We emphasize that the number of studies included in the present meta-analysis was greater than in the other outcomes. Considering that low cardiorespiratory fitness has been explored as a predictor of cardiovascular mortality in people with diabetes [16], the present findings may reflect major clinical benefits. A cohort study, including non-diabetic and diabetic individuals, showed that increments equivalent to 1.44 ml/kg/min in VO_2max_ were associated with a 7.9% reduction in overall mortality [71]. Moreover, subjects with type 1 and 2 diabetes mellitus present lower walking capacity compared with non-diabetic controls [72]. Of note, we observed that in the present synthesis supervised interventions from included studies show an increase of 11% (51.59 m) in the 6MWT, which is considered a reliable, validated, and clinically meaningful test for patients with diabetes [73].

Low muscle strength has been shown to be associated with an increased risk of all-cause mortality [15, 74]. Furthermore, in patients with type 2 diabetes, there is a pronounced decline in muscle mass and strength, in agreement with a worsening in functional performance [4]. Therefore, we can highlight the importance of increases in muscle strength, in addition to the fact that, in response to exercise training, strength improvement might be associated with a lower age-related risk of frailty and sarcopenia [75]. It is also important to highlight the clinical importance of observing increases in functional variables in older individuals after interventions, such as gait and lower-limb strength, for example, due to their negative predictive capacity in relation to the use of health care and adverse events (i.e., institutionalization, falls, disability, mortality) [76–78]. However, it is important to emphasize that the results from our meta-analysis and its estimates related to muscle strength should be interpreted with caution due to the low number of included studies.

To explore the expected methodological and statistical heterogeneity, we used a prespecified strategy based on sensitivity and meta-regression analyses and did not detect associated factors. In addition, the quality of the studies was mostly low, which may have contributed to heterogeneity in the present meta-analyses [30]. Due to the low number of studies available, exploratory analyses were not performed for five of the six intended outcomes, which would require at least 10 studies [30], and for peripheral neuropathy which was not present in any sample. As for analyses with VO_2max_, it was not possible to demonstrate conclusive results due to the occurrence of overlapping confidence intervals, and we did not identify any association between HbA1c and VO_2max_.

Regarding the quality and risk of bias of individual studies, in general, the reporting and internal validity items, the studies obtained good scores on questions such as description of hypothesis/aim, clear description of outcomes and main results, description of variability estimates, number of lost participants, follow-up period for groups. Items of external validity, internal validity—confounding (selection bias) and power were identified as more prone to bias. We emphasize that characteristics contemplating the generalization to the population from which the study participants were derived, adjustment of confounding factors in the analyses, loss of patients in the course of the study and sample size calculation should be considered for the interpretation of results and future studies.

### Limitations

This study has some limitations. Although the search was not limited by language, the studies included were only in Portuguese, English, and Spanish. The clinical conditions that we used as exclusion criteria for the studies were chosen because they strongly influence the functional results, which would end up being a confounding factor and difficult to control for methodologically. We tried to broadly address the functional outcomes in this population; however, within the criteria used to select the studies, some ended up being identified in a low number, thus not being explored as planned. In addition, balance is an important physical parameter and strongly associated with falls; however, we did not evaluate this parameter. We also recognize that our results are based on performance-based measures, which ultimately limit inferences and correlations with self-reported instruments [79]. Finally, we analyzed only structured physical exercise interventions, which may not be feasible for all patients with type 2 diabetes. Therefore, the results presented cannot be generalized to all exercise programs in this population.

Moreover, high heterogeneity was identified in the meta-analyses, especially in the walking performance (6MWT) and physical fitness (VO_2max_) meta-analysis, and although we did try to explore it, no additional information was retrieved with this strategy. However, we did not investigate exercise variables, which could have contributed to a reduction in heterogeneity. Therefore, exploring the types of physical exercise and its specific components (FITT principles—frequency, intensity, time, and type) would be relevant. In addition, the overall quality of the studies was low, increasing the risk of bias in the studies, which may limit the interpretation of results.

### Future Directions

Because many comorbidities are associated with type 2 diabetes, future trials should consider minimizing eligibility criteria to allow more representative samples for this clinical population. Of great is diabetic neuropathy, which is a major comorbidity and a common product of diabetes progression; therefore, we emphasize the importance of future studies clarifying the health status of the participants, thus contributing to the performance of deeper analysis. In addition, establishing common outcomes, such as implementing the use of Core Outcome Set (COS), would be beneficial to increase the number of comparable studies in future reviews [80].

This systematic review demonstrates that structured physical exercise is associated with improvements in functional outcomes with clinical relevance for people with diabetes. This highlights the need and importance of a recommendation for physical exercise in order to preserve and/or improve physical function in this population.

## Conclusions

In conclusion, the current meta-analysis indicates that structured physical exercise programs might improve functional capacity (i.e., cardiorespiratory fitness, walking performance, lower-limb muscle strength, sit and stand up and walk tests) in people with type 2 diabetes. Such increments are more clearly perceived in the VO_2max_ and 6MWT outcomes (as compared to the other outcomes assessed, these two outcomes were the ones that grouped the largest number of studies). However, subgroup and sensitivity analyses were inconclusive due to the small number of studies in some comparison groups and the high variability observed in confidence interval values.

## Supplementary Information

**Additional file 1**. **Appendix 1**. Search strategy; **Appendix 2**. Leave one out with VO_2max_ analysis; **Appendix 3**. Funnel Plot VO_2max_; **Appendix 4**. Quality assessment and of the risk of bias in individual studies assessed by using the Checklist Downs & Black.

## Data Availability

The data and analytic codes used in the meta-analyses and the scripts used to generate the meta-analysis are available with the other materials in the Open Science Framework (OSF) repository, available in: https://osf.io/h47r8/.

## Abbreviations

1RM: One repetition maximum
6MWT: 6-Minute walk test
COS: Core Outcome Set
HbA1c: Glycated hemoglobin
HR%: Heart rate
HRmax: Maximum heart rate
HRpeak: Peak heart rate
HRR: Heart rate reserve
MVC: Maximum voluntary contraction
NRS: Non-randomized controlled studies
OSF: Open Science Framework
PRISMA: Preferred Reporting Items for Systematic Reviews and Meta-Analysis
PRISMA-P: Preferred Reporting Items for Systematic Review and Meta-Analysis Protocols
PROSPERO: International Prospective Register of Systematic Reviews
RCTs: Randomized controlled trials
RM: Repetitions maximum
RPE: Rating of perceived exertion
TUG: Timed Up and Go test
VO2max: Maximal oxygen consumption
VO2peak: Peak oxygen consumption

## References

[1] International Diabetes Federation. IDF Diabetes Atlas Ninth Edition 2019. 2019; Available from: www.diabetesatlas.org 35914061

[2] Reid KF, Doros G, Clark DJ, Patten C, Carabello RJ, Cloutier GJ (2012). Muscle power failure in mobility-limited older adults: preserved single fiber function despite lower whole muscle size, quality and rate of neuromuscular activation. Eur J Appl Physiol.

[3] Volpato S, Bianchi L, Lauretani F, Lauretani F, Bandinelli S, Guralnik JM (2012). Role of muscle mass and muscle quality in the association between diabetes and gait speed. Diabetes Care.

[4] Leenders M, Verdijk LB, van der Hoeven L, Adam JJ, van Kranenburg J, Nilwik R (2013). Patients with type 2 diabetes show a greater decline in muscle mass, muscle strength, and functional capacity with aging. J Am Med Dir Assoc.

[5] Park SW, Goodpaster BH, Strotmeyer ES, Kuller LH, Broudeau R, Kammerer C (2007). Accelerated loss of skeletal muscle strength in older adults with type 2 diabetes: the health, aging, and body composition study. Diabetes Care.

[6] Park SW, Goodpaster BH, Lee JS, Kuller LH, Boudreau R, de Rekeneire N (2009). Excessive loss of skeletal muscle mass in older adults with type 2 diabetes. Diabetes Care.

[7] Aguayo GA, Hulman A, Vaillant MT, Donneau A-F, Schritz A, Stranges S (2019). Prospective association among diabetes diagnosis, HbA1c, glycemia, and frailty trajectories in an elderly population. Diabetes Care.

[8] Wong E, Backholer K, Gearon E, Harding J, Freak-Poli R, Stevenson C (2013). Diabetes and risk of physical disability in adults: a systematic review and meta-analysis. Lancet Diabetes Endocrinol.

[9] Guralnik JM, Branch LG, Cummings SR, Curb JD (1989). Physical performance measures in aging research. J Gerontol.

[10] Kalyani RR, Tra Y, Yeh H-C, Egan JM, Ferrucci L, Brancati FL. Quadriceps strength, quadriceps power, and gait speed in older U.S. adults with diabetes mellitus: results from the National Health and Nutrition Examination Survey, 1999–2002. J Am Geriatr Soc. 2013;61:769–75. 10.1111/jgs.12204 PMC3725774 23617584

[11] Sacchetti M, Balducci S, Bazzucchi I, Carlucci F, Scotto di Palumbo A, Haxhi J, et al. Neuromuscular dysfunction in diabetes: role of nerve impairment and training status. Med Sci Sports Exerc. 2013;45:52–9. 10.1249/MSS.0b013e318269f9bb 22843109

[12] Studenski S, Perera S, Patel K, Rosano C, Faulkner K, Inzitari M (2011). Gait speed and survival in older adults. JAMA.

[13] Guralnik JM, Simonsick EM, Ferrucci L. A short physical performance battery assessing lower extremity function: association with self-reported disability and prediction of mortality and nursing home. Journal of [Internet]. academic.oup.com; 1994; Available from: https://academic.oup.com/geronj/article-abstract/49/2/M85/595537. 10.1093/geronj/49.2.m85 8126356

[14] Bergland A, Jørgensen L, Emaus N, Strand BH. Mobility as a predictor of all-cause mortality in older men and women: 11.8 year follow-up in the Tromsø study. BMC Health Serv Res. 2017;17:22. 10.1186/s12913-016-1950-0 PMC5223479 28068995

[15] Li R, Xia J, Zhang XI, Gathirua-Mwangi WG, Guo J, Li Y (2018). Associations of muscle mass and strength with all-cause mortality among US older adults. Med Sci Sports Exerc.

[16] Wei M, Gibbons LW, Kampert JB, Nichaman MZ, Blair SN (2000). Low cardiorespiratory fitness and physical inactivity as predictors of mortality in men with type 2 diabetes. Ann Intern Med.

[17] Wray LA, Ofstedal MB, Langa KM, Blaum CS (2005). The effect of diabetes on disability in middle-aged and older adults. J Gerontol A Biol Sci Med Sci.

[18] Chiu C-J, Wray LA, Ofstedal MB (2011). Diabetes-related change in physical disability from midlife to older adulthood: evidence from 1996–2003 Survey of Health and Living Status of the Elderly in Taiwan. Diabetes Res Clin Pract.

[19] Kyrou I, Tsigos C, Mavrogianni C, Cardon G, Van Stappen V, Latomme J (2020). Sociodemographic and lifestyle-related risk factors for identifying vulnerable groups for type 2 diabetes: a narrative review with emphasis on data from Europe. BMC Endocr Disord.

[20] Alvarado BE, Zunzunegui M-V, Béland F, Bamvita J-M (2008). Life course social and health conditions linked to frailty in Latin American older men and women. J Gerontol A Biol Sci Med Sci.

[21] Makizako H, Shimada H, Doi T, Tsutsumimoto K, Hotta R, Nakakubo S, et al. Social frailty leads to the development of physical frailty among physically non-frail adults: a four-year follow-up longitudinal cohort study. Int J Environ Res Public Health. 2018;15. 10.3390/ijerph15030490. 10.3390/ijerph15030490 PMC5877035 29534470

[22] Colberg SR, Sigal RJ, Yardley JE, Riddell MC, Dunstan DW, Dempsey PC (2016). Physical activity/exercise and diabetes: a position statement of the American Diabetes Association. Diabetes Care.

[23] Gregg EW, Lin J, Bardenheier B, Chen H, Rejeski WJ, Zhuo X (2018). Impact of intensive lifestyle intervention on disability-free life expectancy: the Look AHEAD Study. Diabetes Care.

[24] Balducci S, Zanuso S, Cardelli P, Salvi L, Mazzitelli G, Bazuro A (2012). Changes in physical fitness predict improvements in modifiable cardiovascular risk factors independently of body weight loss in subjects with type 2 diabetes participating in the Italian Diabetes and Exercise Study (IDES). Diabetes Care.

[25] Houston DK, Leng X, Bray GA, Hergenroeder AL, Hill JO, Jakicic JM (2015). A long-term intensive lifestyle intervention and physical function: the look AHEAD Movement and Memory Study. Obesity.

[26] Jakicic JM, Jaramillo SA, Balasubramanyam A, Bancroft B, Curtis JM, Mathews A (2009). Effect of a lifestyle intervention on change in cardiorespiratory fitness in adults with type 2 diabetes: results from the Look AHEAD Study. Int J Obes.

[27] Brandon LJ, Gaasch DA, Boyette LW, Lloyd AM (2003). Effects of long-term resistive training on mobility and strength in older adults with diabetes. J Gerontol A Biol Sci Med Sci.

[28] Botton CE, Umpierre D, Rech A, Pfeifer LO, Machado CLF, Teodoro JL (2018). Effects of resistance training on neuromuscular parameters in elderly with type 2 diabetes mellitus: A randomized clinical trial. Exp Gerontol.

[29] Page MJ, McKenzie JE, Bossuyt PM, Boutron I, Hoffmann TC, Mulrow CD, et al. The PRISMA 2020 statement: an updated guideline for reporting systematic reviews. BMJ. 2021;372:n71. 10.1136/bmj.n71 PMC8005924 33782057

[30] Higgins JPT, Thomas J, Chandler J, Cumpston M, Li T, Page MJ, et al. Cochrane handbook for systematic reviews of interventions. John Wiley & Sons; 2019. 10.1002/14651858.ED000142 PMC10284251 31643080

[31] Moher D, Shamseer L, Clarke M, Ghersi D, Liberati A, Petticrew M (2015). Preferred reporting items for systematic review and meta-analysis protocols (PRISMA-P) 2015 statement. Syst Rev.

[32] Downs SH, Black N (1998). The feasibility of creating a checklist for the assessment of the methodological quality both of randomised and non-randomised studies of health care interventions. J Epidemiol Community Health.

[33] Huffer D, Hing W, Newton R, Clair M (2017). Strength training for plantar fasciitis and the intrinsic foot musculature: a systematic review. Phys Ther Sport.

[34] Spineli LM, Pandis N (2020). Prediction interval in random-effects meta-analysis. Am J Orthod Dentofacial Orthop.

[35] Pfeifer LO, De Nardi AT, da Silva LXN, Nascimento DM do, Botton CE, Teodoro JL, et al. Association between physical activity interventions and functional capacity in middle-aged adults and older individuals with type 2 diabetes: a protocol for a systematic review and meta-analysis of randomized or non-randomized clinical trials [Internet]. 2020. https://osf.io/kpg2m.

[36] Shabkhiz F, Khalafi M, Rosenkranz S, Karimi P, Moghadami K. Resistance training attenuates circulating FGF-21 and myostatin and improves insulin resistance in elderly men with and without type 2 diabetes mellitus: a randomised controlled clinical trial. EJSS . Informa UK Limited; 2020;1–10. 10.1080/17461391.2020.1762755 32345132

[37] Yamamoto Y, Nagai Y, Kawanabe S, Hishida Y, Hiraki K, Sone M (2021). Effects of resistance training using elastic bands on muscle strength with or without a leucine supplement for 48 weeks in elderly patients with type 2 diabetes. Endocr J.

[38] Conners RT, Caputo JL, Coons JM, Fuller DK, Morgan DW (2019). Impact of underwater treadmill training on glycemic control, blood lipids, and health-related fitness in adults with type 2 diabetes. Clin Diabetes.

[39] Hwang C-L, Lim J, Yoo J-K, Kim H-K, Hwang M-H, Handberg EM, et al. Effect of all-extremity high-intensity interval training vs. moderate-intensity continuous training on aerobic fitness in middle-aged and older adults with type 2 diabetes: a randomized controlled trial. Exp Gerontol. 2019;116:46–53. 10.1016/j.exger.2018.12.013 PMC6404965 30576716

[40] Szilágyi B, Kukla A, Makai A, Ács P, Járomi M (2019). Sports therapy and recreation exercise program in type 2 diabetes: randomized controlled trial, 3-month follow-up. J Sports Med Phys Fitness.

[41] Melo KCB, Araújo F de S, Cordeiro Júnior CCM, de Andrade KTP, Moreira SR. Pilates method training: functional and blood glucose responses of older women with type 2 diabetes. J Strength Cond Res. 2020;34:1001–7. 10.1519/JSC.0000000000002704 29985228

[42] del Pozo-Cruz B, Alfonso-Rosa RM, del Pozo-Cruz J, Sañudo B, Rogers ME (2014). Effects of a 12-wk whole-body vibration based intervention to improve type 2 diabetes. Maturitas.

[43] Tan S, Li W, Wang J (2012). Effects of six months of combined aerobic and resistance training for elderly patients with a long history of type 2 diabetes. J Sports Sci Med.

[44] Balducci S, Zanuso S, Nicolucci A, Fernando F, Cavallo S, Cardelli P (2010). Anti-inflammatory effect of exercise training in subjects with type 2 diabetes and the metabolic syndrome is dependent on exercise modalities and independent of weight loss. Nutr Metab Cardiovasc Dis.

[45] Lam P, Dennis SM, Diamond TH, Zwar N. Improving glycaemic and BP control in type 2 diabetes. The effectiveness of tai chi. Aust Fam Physician. 2008;37:884–7. 19002314

[46] Bjørgaas M, Vik JT, Saeterhaug A, Langlo L, Sakshaug T, Mohus RM (2005). Relationship between pedometer-registered activity, aerobic capacity and self-reported activity and fitness in patients with type 2 diabetes. Diabetes Obes Metab Wiley.

[47] Jiang Y, Tan S, Wang Z, Guo Z, Li Q, Wang J (2020). Aerobic exercise training at maximal fat oxidation intensity improves body composition, glycemic control, and physical capacity in older people with type 2 diabetes. J Exerc Sci Fit.

[48] Labrunée M, Antoine D, Vergès B, Robin I, Casillas J-M, Gremeaux V (2012). Effects of a home-based rehabilitation program in obese type 2 diabetics. Ann Phys Rehabil Med.

[49] Brun J-F, Bordenave S, Mercier J, Jaussent A, Picot M-C, Préfaut C (2008). Cost-sparing effect of twice-weekly targeted endurance training in type 2 diabetics: a one-year controlled randomized trial. Diabetes Metab.

[50] Larose J, Sigal RJ, Boulé NG, Wells GA, Prud’homme D, Fortier MS, et al. Effect of exercise training on physical fitness in type II diabetes mellitus [Internet]. Med Sci Sports Exer. 2010. p. 1439–47. 10.1249/mss.0b013e3181d322dd 10.1249/MSS.0b013e3181d322dd 20639722

[51] Loimaala A, Huikuri HV, Kööbi T, Rinne M, Nenonen A, Vuori I (2003). Exercise training improves baroreflex sensitivity in type 2 diabetes. Diabetes.

[52] Karstoft K, Winding K, Knudsen SH, Nielsen JS, Thomsen C, Pedersen BK (2013). The effects of free-living interval-walking training on glycemic control, body composition, and physical fitness in type 2 diabetic patients: a randomized, controlled trial. Diabetes Care.

[53] Kadoglou NPE, Iliadis F, Angelopoulou N, Perrea D, Ampatzidis G, Liapis CD (2007). The anti-inflammatory effects of exercise training in patients with type 2 diabetes mellitus. Eur J Cardiovasc Prev Rehabil.

[54] Kadoglou NPE, Iliadis F, Sailer N, Athanasiadou Z, Vitta I, Kapelouzou A (2010). Exercise training ameliorates the effects of rosiglitazone on traditional and novel cardiovascular risk factors in patients with type 2 diabetes mellitus. Metabolism.

[55] Loimaala A, Groundstroem K, Rinne M, Nenonen A, Huhtala H, Parkkari J (2009). Effect of long-term endurance and strength training on metabolic control and arterial elasticity in patients with type 2 diabetes mellitus. Am J Cardiol.

[56] Verity LS, Ismail AH (1989). Effects of exercise on cardiovascular disease risk in women with NIDDM. Diabetes Res Clin Pract.

[57] Plotnikoff RC, Eves N, Jung M, Sigal RJ, Padwal R, Karunamuni N (2010). Multicomponent, home-based resistance training for obese adults with type 2 diabetes: a randomized controlled trial. Int J Obes.

[58] Yan H, Prista A, Ranadive SM, Damasceno A, Caupers P, Kanaley JA, et al. Effect of aerobic training on glucose control and blood pressure in T2DDM East African males. ISRN Endocrinol. 2014;2014:864897. 10.1155/2014/864897 PMC3960729 24729886

[59] Banitalebi E, Kazemi A, Faramarzi M, Nasiri S, Haghighi MM (2019). Effects of sprint interval or combined aerobic and resistance training on myokines in overweight women with type 2 diabetes: A randomized controlled trial. Life Sci.

[60] Wilson GA, Wilkins GT, Cotter JD, Lamberts RR, Lal S, Baldi JC (2019). HIIT improves left ventricular exercise response in adults with type 2 diabetes. Med Sci Sports Exerc.

[61] Scheer AS, Naylor LH, Gan SK, Charlesworth J, Benjanuvatra N, Green DJ (2020). The effects of water-based exercise training in people with type 2 diabetes. Med Sci Sports Exerc.

[62] dos Santos GM, Montrezol FT, Pauli LSS, Sartori-Cintra AR, Colantonio E, Gomes RJ (2014). Undulatory physical resistance training program increases maximal strength in elderly type 2 diabetics. Einstein.

[63] Fritz T, Wändell P, Åberg H, Engfeldt P (2006). Walking for exercise—Does three times per week influence risk factors in type 2 diabetes?. Diabetes Res Clin Pract.

[64] Skarfors ET, Wegener TA, Lithell H, Selinus I. Physical training as treatment for type 2 (non-insulin-dependent) diabetes in elderly men. A feasibility study over 2 years. Diabetologia. 1987;30:930–3. 10.1007/BF00295876 3436489

[65] Magalhães JP, Júdice PB, Ribeiro R, Andrade R, Raposo J, Dores H (2019). Effectiveness of high-intensity interval training combined with resistance training versus continuous moderate-intensity training combined with resistance training in patients with type 2 diabetes: A one-year randomized controlled trial. Diabetes Obes Metab.

[66] Stubbs EB, Fisher MA, Miller CM, Jelinek C, Butler J, McBurney C (2019). Randomized controlled trial of physical exercise in diabetic veterans with length-dependent distal symmetric polyneuropathy. Front Neurosci.

[67] Pozo-Cruz J del, del Pozo-Cruz J, Alfonso-Rosa RM, Ugia JL, McVeigh JG, del Pozo-Cruz B, et al. A primary care–based randomized controlled trial of 12-week whole-body vibration for balance improvement in type 2 diabetes mellitus. Arch Phys Med Rehabil. 2013. p. 2112–8. 10.1016/j.apmr.2013.05.030. 10.1016/j.apmr.2013.05.030 23811317

[68] Otten J, Stomby A, Waling M, Isaksson A, Tellström A, Lundin-Olsson L, et al. Benefits of a Paleolithic diet with and without supervised exercise on fat mass, insulin sensitivity, and glycemic control: a randomized controlled trial in individuals with type 2 diabetes. Diabetes Metab Res Rev. 2017;33. 10.1002/dmrr.2828 10.1002/dmrr.2828 PMC5402870 27235022

[69] Boulé NG, Kenny GP, Haddad E, Wells GA, Sigal RJ (2003). Meta-analysis of the effect of structured exercise training on cardiorespiratory fitness in Type 2 diabetes mellitus. Diabetologia.

[70] Yang Z, Scott CA, Mao C, Tang J, Farmer AJ (2014). Resistance exercise versus aerobic exercise for type 2 diabetes: a systematic review and meta-analysis. Sports Med.

[71] Blair SN. Changes in physical fitness and all-cause mortality. JAMA. 1995. p. 1093. 10.1001/jama.1995.03520380029031. 7707596

[72] Kuziemski K, Słomiński W, Jassem E (2019). Impact of diabetes mellitus on functional exercise capacity and pulmonary functions in patients with diabetes and healthy persons. BMC Endocr Disord.

[73] Lee MC (2018). Validity of the 6-minute walk test and step test for evaluation of cardio respiratory fitness in patients with type 2 diabetes mellitus. J Exerc Nutr Biochem.

[74] Newman AB, Kupelian V, Visser M, Simonsick EM, Goodpaster BH, Kritchevsky SB (2006). Strength, but not muscle mass, is associated with mortality in the health, aging and body composition study cohort. J Gerontol A Biol Sci Med Sci.

[75] Izquierdo M, Merchant RA, Morley JE, Anker SD, Aprahamian I, Arai H, et al. International exercise recommendations in older adults (ICFSR): expert consensus guidelines. J Nutr Health Aging. 2021. 10.1007/s12603-021-1665-8.10.1007/s12603-021-1665-834409961

[76] Abellan van Kan G, Rolland Y, Andrieu S, Bauer J, Beauchet O, Bonnefoy M, et al. Gait speed at usual pace as a predictor of adverse outcomes in community-dwelling older people an International Academy on Nutrition and Aging (IANA) Task Force. J Nutr Health Aging. 2009;13:881–9.10.1007/s12603-009-0246-z19924348

[77] Cesari M, Kritchevsky SB, Newman AB, Simonsick EM, Harris TB, Penninx BW (2009). Added value of physical performance measures in predicting adverse health-related events: results from the Health, Aging And Body Composition Study. J Am Geriatr Soc.

[78] Studenski S, Perera S, Wallace D, Chandler JM, Duncan PW, Rooney E (2003). Physical performance measures in the clinical setting. J Am Geriatr Soc.

[79] Onodera CMK, Coelho-Júnior HJ, Sampaio RAC, Santos Duarte Lana JF, Teixeira LFM, Uchida MC, et al. The importance of objectively measuring functional tests in complement to self-report assessments in patients with knee osteoarthritis. Gait Posture. 2020;82:33–7. 10.1016/j.gaitpost.2020.08.121 32871410

[80] Harman NL, Wilding JPH, Curry D, Harris J, Logue J, Pemberton RJ, et al. Selecting Core Outcomes for Randomised Effectiveness trials In Type 2 diabetes (SCORE-IT): a patient and healthcare professional consensus on a core outcome set for type 2 diabetes. BMJ Open Diabetes Res Care. 2019;7:e000700. 10.1136/bmjdrc-2019-000700 PMC6936506 31908789

